# Supplementary material for: Social influences on delayed gratification in New Caledonian crows and Eurasian jays
Source: PLoS One. 2023 Dec 6;18(12):e0289197. doi: 10.1371/journal.pone.0289197 (PMC10699590; doi:10.1371/journal.pone.0289197)
Supplement: S1 Table — *Change in relative dominance between sessions (see S2 Table). (DOCX) [file pone.0289197.s001.docx]

**Social influences on delayed gratification in New Caledonian crows and Eurasian jays**

Rachael Miller, James R. Davies, Martina Schiestl, Elias Garcia-Pelegrin, Russell D. Gray, Alex H. Taylor, Nicola S. Clayton

**Supporting Information**

**S1 Table: Subject Information.** *Change in relative dominance between sessions (see S2 Table).

| **Subject** | **Species** | **Caught with (NC crows only)** | **Sex** | **Age** | **Competitor** | **Non-competitor** |
| --- | --- | --- | --- | --- | --- | --- |
| Leaky | NC crows | Fossey, Birute | M | adult | Fossey | Birute |
| Fossey | NC crows | Leaky, Birute | M | 1st year | Leakey | Birute |
| Birute | NC crows | Leaky, Fossey | F | 1st year | Fossey | Leakey |
| Konrad | NC crows | Irene, Marie | M | 2nd year | Irene | Marie |
| Marie | NC crows | Konrad, Irene | F | 1st year | Konrad | Irene |
| Irene | NC crows | Marie, Konrad | F | 2nd year | Konrad | Marie |
| Godot | E jays | / | M | adult | Homer | Jaylo, Booster |
| Homer | E jays | / | M | adult | Horatio | Poe, Stuka, Lintie |
| Penny | E jays | / | F | adult | Stuka* | Godot, Booster, Stuka* |
| Sjoika | E jays | / | F | adult | Stuka, Homer | Godot |
| Stuka | E jays | / | F | adult | Dolci, Poe, Homer | Sjoika, Penny, Jaylo, Dexter |
