# Supplementary material for: Social influences on delayed gratification in New Caledonian crows and Eurasian jays
Source: PLoS One. 2023 Dec 6;18(12):e0289197. doi: 10.1371/journal.pone.0289197 (PMC10699590; doi:10.1371/journal.pone.0289197)
Supplement: S2 Table — *Change in relative dominance between two specific individuals. (DOCX) [file pone.0289197.s002.docx]

**Social influences on delayed gratification in New Caledonian crows and Eurasian jays**

Rachael Miller, James R. Davies, Martina Schiestl, Elias Garcia-Pelegrin, Russell D. Gray, Alex H. Taylor, Nicola S. Clayton

**Supporting Information**

**S2 Table: Food monopolization results for the Eurasian jays.** *Change in relative dominance between two specific individuals.

| **Date** | **Focal Bird** | **Other** | **Dominant (competitor)** |
| --- | --- | --- | --- |
| 15/11/2022 | Booster | Penny | Penny |
| 15/11/2022 | Homer | Jaylo | Homer |
| 15/11/2022 | Homer | Sojka | Sojka |
| 15/11/2022 | Jaylo | Penny | Jaylo |
| 15/11/2022 | Jaylo | Stuka | Jaylo |
| 15/11/2022 | Penny | Homer | Homer |
| 15/11/2022 | Penny | Sojka | Sojka |
| 15/11/2022 | Poe | Homer | Homer |
| 15/11/2022 | Poe | Jaylo | Jaylo |
| 15/11/2022 | Poe | Penny | Poe |
| 15/11/2022 | Sojka | Poe | Sojka |
| 15/11/2022 | Sojka | Stuka | Sojka |
| 15/11/2022 | Stuka | Jaylo | Jaylo |
| 15/11/2022 | Stuka | Penny | Penny |
| 15/11/2022 | Stuka | Sojka | Sojka |
| 18/11/2022 | Homer | Booster | Homer |
| 18/11/2022 | Sojka | Poe | Sojka |
| 18/11/2022 | Stuka | Homer | Homer |
| 18/11/2022 | Stuka | Sojka | Sojka |
| 21/11/2022 | Sojka | Jaylo | Sojka |
| 06/12/2022 | Sojka | Stuka | Stuka* |
| 08/12/2022 | Penny | Stuka | Penny |
| 13/01/2023 | Poe | Homer | Homer |
| 17/02/2023 | Penny | Stuka | Stuka* |
| 22/02/2023 | Penny | Godot | Penny |
| 23/02/2023 | Sojka | Godot | Sojka |
| 24/02/2023 | Stuka | Poe | Poe |
| 28/02/2023 | Booster | Penny | Penny |
| 07/03/2023 | Stuka | Homer | Homer |
| 23/03/2023 | Jaylo | Stuka | Stuka* |
| 24/03/2023 | Sojka | Homer | Homer* |
| 24/03/2023 | Stuka | Dexter | Stuka |
| 29/03/2023 | Penny | Sojka | Sojka |
| 13/04/2023 | Homer | Lintie | Homer |
| 14/04/2023 | Sojka | Lintie | Lintie |
| 24/04/2023 | Godot | Jaylo | Godot |
| 25/04/2023 | Godot | Homer | Homer |
| 03/05/2023 | Booster | Godot | Godot |
