# Supplementary material for: Social influences on delayed gratification in New Caledonian crows and Eurasian jays
Source: PLoS One. 2023 Dec 6;18(12):e0289197. doi: 10.1371/journal.pone.0289197 (PMC10699590; doi:10.1371/journal.pone.0289197)
Supplement: S3 Table — * = did not reach criterion: three of eight jays did not reach criterion within 15 sessions (*) so were excluded from further testing. NC crows = New Caledonian crows; E jays = Eurasian jays. (DOCX) [file pone.0289197.s003.docx]

**Social influences on delayed gratification in New Caledonian crows and Eurasian jays**

Rachael Miller, James R. Davies, Martina Schiestl, Elias Garcia-Pelegrin, Russell D. Gray, Alex H. Taylor, Nicola S. Clayton

**Supporting Information**

**S3 Table. ‘Learning speed’ per individual and species: number of trials and sessions to reach criterion and complete test trials (last 2 sessions counted) in alone condition.** * = did not reach criterion: three of eight jays did not reach criterion within 15 sessions (*) so were excluded from further testing. NC crows = New Caledonian crows; E jays = Eurasian jays.

| **Subject** | **Species** | **No. of trials overall** | **No. of sessions** |
| --- | --- | --- | --- |
| Birute | NC crows | 20 | 2 |
| Fossey | NC crows | 20 | 2 |
| Irene | NC crows | 20 | 2 |
| Konrad | NC crows | 20 | 2 |
| Leakey | NC crows | 20 | 2 |
| Marie | NC crows | 20 | 2 |
| Godot | E jays | 55 | 6 |
| Homer | E jays | 50 | 5 |
| Penny | E jays | 70 | 7 |
| Sjoika | E jays | 30 | 3 |
| Stuka | E jays | 80 | 8 |
| *Booster | E jays | 138 | 15 |
| *Poe | E jays | 147 | 15 |
| *Jaylo | E jays | 150 | 15 |
