# Supplementary material for: Social influences on delayed gratification in New Caledonian crows and Eurasian jays
Source: PLoS One. 2023 Dec 6;18(12):e0289197. doi: 10.1371/journal.pone.0289197 (PMC10699590; doi:10.1371/journal.pone.0289197)

**Social influences on delayed gratification in New Caledonian crows and Eurasian jays**

Rachael Miller, James R. Davies, Martina Schiestl, Elias Garcia-Pelegrin, Russell D. Gray, Alex H. Taylor, Nicola S. Clayton

**Supporting Information**

**S4 Figure. Timeline of pretraining and testing phases**. Coloured arrows show the different test condition sequences that individuals were assigned to for sessions one through to four (S1-S4). The dotted line shows a possible repeat of the food monopolization phase if relative dominance relationships were perceived to change mid-test sequence (jays only).


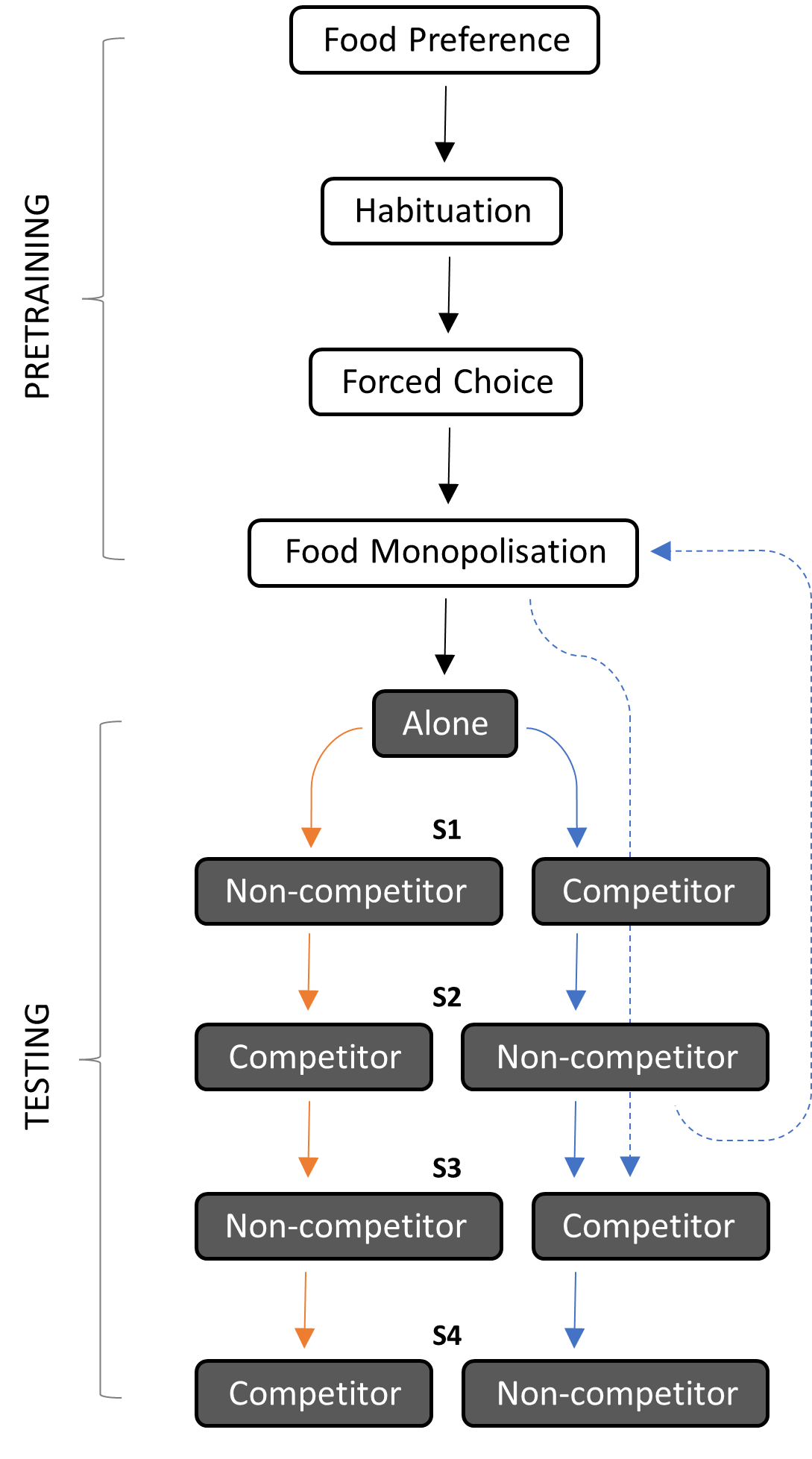

Supplement: S1 Fig — Coloured arrows show the different test condition sequences that individuals were assigned to for sessions one through to four (S1-S4). The dotted line shows a possible repeat of the food monopolization phase if relative dominance relationships were perceived to change mid-test sequence (jays only). (DOCX) [file pone.0289197.s004.docx]
