# Supplementary material for: Social influences on delayed gratification in New Caledonian crows and Eurasian jays
Source: PLoS One. 2023 Dec 6;18(12):e0289197. doi: 10.1371/journal.pone.0289197 (PMC10699590; doi:10.1371/journal.pone.0289197)
Supplement: S1 File — (DOCX) [file pone.0289197.s005.docx]

**Social influences on delayed gratification in New Caledonian crows and Eurasian jays**

Rachael Miller, James R. Davies, Martina Schiestl, Elias Garcia-Pelegrin, Russell D. Gray, Alex H. Taylor, Nicola S. Clayton

**Supporting Information**

**S5 Resource. Example video trials for both species**

[**https://www.youtube.com/watch?v=bJ2ubS4yPtM&ab_channel=JamesDavies**](https://www.youtube.com/watch?v=bJ2ubS4yPtM&ab_channel=JamesDavies)
