# Supplementary material for: Social influences on delayed gratification in New Caledonian crows and Eurasian jays
Source: PLoS One. 2023 Dec 6;18(12):e0289197. doi: 10.1371/journal.pone.0289197 (PMC10699590; doi:10.1371/journal.pone.0289197)
Supplement: S2 File — (DOCX) [file pone.0289197.s006.docx]

**Social influences on delayed gratification in New Caledonian crows and Eurasian jays**

Rachael Miller, James R. Davies, Martina Schiestl, Elias Garcia-Pelegrin, Russell D. Gray, Alex H. Taylor, Nicola S. Clayton

**Supporting Information**

**S6 Resource. Comparison of baseline to Miller et al. 2020 [55]**

In Miller et al (2020), using the rotating-tray paradigm with a different sample of New Caledonian crows than the present study, we found that eight of nine NC crows tested passed a comparable condition, i.e., selected the high-quality, delayed reward over the low-quality, immediate reward while alone with rewards varying in quality (Individual-level performance: Binomial exact two-tailed tests: p < 0.001).
